# Clinical characteristics and risk factors for cefaclor-induced immediate hypersensitivity: a retrospective observation at two university hospitals in Korea

**DOI:** 10.1186/s13223-021-00523-8

**Published:** 2021-02-15

**Authors:** Hyo-In Rhyou, Go-Eun Doo, Jiwon Yoon, Chae-Yeon Ha, Hee-Joo Nam, Sung-Dae Woo, Youngsoo Lee, Young-Hee Nam, Young-Min Ye

**Affiliations:** 1 Department of Internal Medicine, College of Medicine, Dong-A University, 26 Daesingongwon-ro, Seo-Gu, Busan, Korea; 2 Dong-A Regional Pharmacovigilance Center, Dong-A University Hospital, 26 Daesingongwon-ro, Seo-Gu, Busan, Korea; 3 Ajou Regional Pharmacovigilance Center, Ajou University Hospital, 164 Worldcup-ro, Yeongtong-gu, Suwon, 443-721 Korea; 4 Department of Pharmacy, Dong-A University Hospital, 26 Daesingongwon-ro, Seo-Gu, Busan, Korea; 5 Department of Allergy and Clinical Immunology, Ajou University School of Medicine, 164 Worldcup-ro, Yeongtong-gu, Suwon, 443-721 Korea

**Keywords:** Cefaclor, Hypersensitivity, Immediate, Clinical characteristics, Risk factor, Incidence

## Abstract

**Background:**

Cefaclor, a second-generation oral cephalosporin, is widely prescribed to treat infectious diseases. Immediate hypersensitivity (HS) reactions to cefaclor have continuously been reported and are expected to increase with its greater use. This study aimed to investigate the clinical characteristics and risk factors of immediate HS to cefaclor over the most recent 5 years.

**Methods:**

This retrospective study investigated 521 adverse drug reactions (ADRs) to cefaclor at pharmacovigilance centers at two tertiary hospitals from January 2014 to December 2018. In total, 459 patients with immediate HS to cefaclor were reviewed.

**Results:**

A total of 459 cases of cefaclor immediate HS were included among 521 cefaclor ADRs, and anaphylaxis was recorded in 61.2%. Female sex (odds ratio 2.917, 95% confidence interval 2.397–3.550, *P* < 0.001), age under 65 years (4.225, 3.017–5.916, *P* < 0.001), hypertension (2.520, 1.875–3.388, *P* < 0.001), liver diseases (2.189, 1.208–3.967, *P* = 0.010), asthma (8.075, 5.301–12.302, *P* < 0.001), and concomitant use of nonsteroidal anti-inflammatory drugs (1.888, 1.554–2.294, *P* < 0.001) were significantly associated with cefaclor immediate HS.

**Conclusions:**

Cefaclor was found to elicit high proportions of immediate HS and anaphylaxis. Physicians ought to be cautious with prescribing cefaclor to females, individuals with hypertension, liver diseases, or asthma, and patients taking nonsteroidal anti-inflammatory drugs.

**Trial registration:**

This study was retrospectively registered.

## Background

The World Health Organization defines an adverse drug reaction (ADR) as a noxious and unintended response to a drug that occurs at doses normally used for prophylaxis, diagnosis, and treating a disease or for modifying physiological function [1]. ADRs are commonly classified as type A or type B: type A reactions can be predicted from the known pharmacology of a drug, whereas type B reactions are idiosyncratic and cannot be predicted from the known pharmacology of a drug [1, 2]. Drug hypersensitivity, a type B reaction, can be subdivided into immediate and non-immediate reactions, depending on the latent period between drug exposure and ADR onset [3]. Drug hypersensitivity is an uncommon, unpredictable, and potentially fatal reaction, especially in cases of anaphylaxis [4]. While research on drug hypersensitivity is ongoing, data on the prevalence, incidence, and risk factors for drug hypersensitivity are lacking.

Cefaclor is a second-generation oral cephalosporin used to treat various infectious diseases [5, 6]. The prescription patterns of antibiotics vary greatly by region and country. Cefaclor was not listed as a commonly prescribed antibiotic in recent years in United states [7–10]. However, according to Health Insurance Review and Assessment Service data, the prescription of cefaclor has continued to increase since 2015 in Korea [11], and therefore, an increase in ADRs related to cefaclor is expected: cefaclor ADRs have been reported continuously [5, 12–17], and cefaclor has been found to be the most common causative drug of anaphylaxis [18]. However, there has been no large-scale study of cefaclor immediate hypersensitivity (HS) and/or anaphylaxis. In this study, we investigated clinical characteristics and risk factors of cefaclor immediate HS, including anaphylaxis, for the most recent 5 years in Korea.

## Materials and methods

### Study subjects and materials

**Fig. 1:**
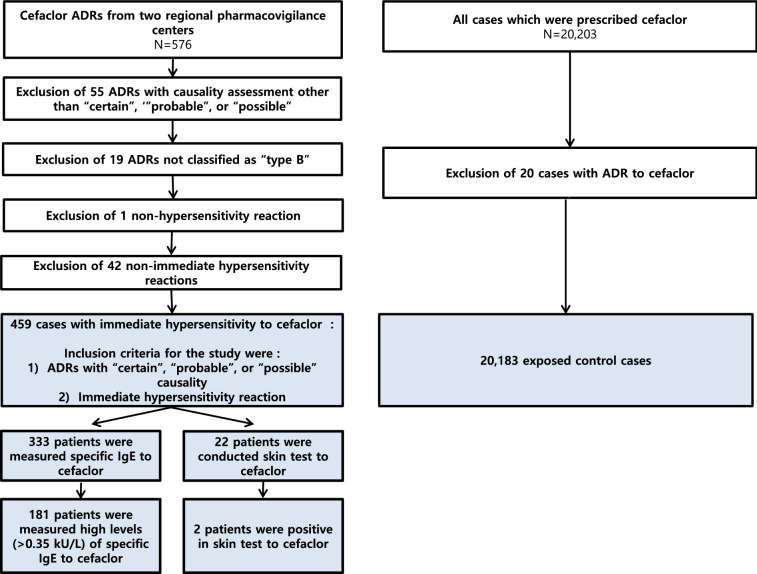
Selection of cases for study

For this retrospective study, all cases of spontaneously reported ADRs to cefaclor were collected from the regional pharmacovigilance centers at two tertiary hospitals in Korea from January 2014 to December 2018. Both pharmacovigilance centers follow the same standard operating policies stipulated by the Korean Food and Drug Administration. Through a spontaneous reporting system, all healthcare persons, such as physicians, nurses, pharmacists, and technicians, as well as patients or their caregivers, are able to report ADRs in both inpatient and outpatient settings to the two pharmacovigilance centers. ADRs were subdivided into type A and type B reactions as previously described, and type B reactions primarily included HSs that were subdivided into immediate HS and delayed HS [1, 2]. Immediate HS was defined as an event that occurred within 1 h following administration of the culprit drug and appeared as urticaria, angioedema, rhinitis, conjunctivitis, bronchospasm, or anaphylaxis. Delayed (or non-immediate) HS was defined as an event that occurred at any time from 1 h after administration of the culprit drug and appeared as delayed-appearing urticaria and/or angioedema, maculopapular exanthema, or severe cutaneous adverse reactions [3, 19]. In addition, anaphylaxis was diagnosed according to diagnostic criteria set forth in the 2011 World Allergy Organization Anaphylaxis Guidelines [20]. In this study, the causality and types of ADRs were evaluated in two steps: trained nurses and pharmacists first evaluated the ADRs, after which expert allergists at both pharmacovigilance centers conducted a final review. If any discrepancy was found in the assessment of ADRs, all reviewers discussed and made a final decision. We included cases in which cefaclor had been prescribed at one of the two hospitals during the study period, but no cefaclor-associated ADRs were detected by the pharmacovigilance centers in the exposed control group (Fig. 1). As mentioned above, the causality of ADRs was assessed using the World Health Organization-Uppsala Monitoring Center criteria, and ADRs of possible, probable, or certain cause were included in the present study (Table 1) [21].

**Table 1:** WHO-UMC causality categories

Causality term	Assessment criteria^a^
Certain	Event or laboratory test abnormality, with plausible time relationship to drug intake Cannot be explained by disease or other drugs Response to withdrawal plausible (pharmacologically, pathologically) Event definitive pharmacologically or phenomenologically (i.e., an objective and specific medical disorder or a recognized pharmacological phenomenon) Rechallenge satisfactory, if necessary
Probable/likely	Event or laboratory test abnormality, with reasonable time relationship to drug intake Unlikely to be attributed to disease or other drugs Response to withdrawal clinically reasonable Rechallenge not required
Possible	Event or laboratory test abnormality, with reasonable time relationship to drug intake Could also be explained by disease or other drugs Information on drug withdrawal may be lacking or unclear
Unlikely	Event of laboratory test abnormality, with a time to drug intake that makes a relationship improbable (but not impossible) Disease or other drugs provide plausible explanations
Conditional/unclassified	Event or laboratory test abnormality More data for proper assessment needed, or Additional data under examination
Unassessable/unclassifiable	Report suggesting an adverse reaction Cannot be judged because information is insufficient or contradictory Data cannot be supplemented or verified

^a^All points should be reasonably complied with

In addition, we analyzed clinical characteristics, including age, sex, underlying disease, concomitant medications, and allergic disease, of the cases of ADRs to cefaclor and the exposed control cases from a review of electronic medical records. Underlying and allergic diseases were identified using the 9th version of the Korean Standard Classification of Diseases (KCD) codes from the two hospitals’ records in June 2019. Atopy was determined by allergen skin prick test. Wheals of a mean diameter ≥ 3 mm for any allergen in skin prick tests to 49 common inhalant allergens were considered as indicative of atopy (Additional file 1: S1 Description). This study was approved by the institutional review boards of both hospitals (AJIRB-MED-MDB-19-231 and DAUHIRB-19-136).

### Allergic evaluation of cefaclor ADRs

#### Serum specific IgE

Serum specific IgE levels to cefaclor were analyzed using ImmunoCAP (Thermo Fisher Scientific) in patients with cefaclor immediate HS. The levels of serum tryptase, total IgE, and specific IgEs to penicilloyl G, penicilloyl V, ampicilloyl, and amoxicilloyl were also analyzed. The cut off for a positive result for the ImmunoCAP was 0.35 kU/L.

#### Skin and oral provocation tests

Skin tests were conducted for a small sample of individuals with suspected cefaclor-induced immediate HS, but who had negative results for serum specific IgE to cefaclor. Skin prick tests were given priority, and for negative on prick tests, intradermal tests were performed. Test solutions were always freshly prepared. Skin prick and intradermal test substances were prepared at increasing concentrations of 0.1 mg/mL, 1 mg/mL, and 10 mg/mL. Skin tests were performed from the lowest concentration, and when a negative result was obtained after 20 min, the tests were sequentially performed at a higher concentration. Oral provocation tests were conducted for individuals with negative results on skin tests to cefaclor after obtaining informed consent. Oral provocation tests were conducted in the order of placebo and 62.5 mg, 125 mg, and 250 mg of cefaclor. If the result was negative 30 min after taking the drug, the next step was sequentially performed. When anaphylaxis or laryngeal edema occurred, epinephrine and systemic steroid were administered immediately. Antihistamine was administered to patients with urticaria. Both the skin and oral provocation tests were performed 1 month after the occurrence of a drug hypersensitivity reaction.

### Statistical analysis

Statistical analyses were conducted using IBM SPSS, version 25 for Windows (IBM SPSS Inc., Chicago, IL, USA). Categorical variables are described as frequencies and proportions, and continuous variables are presented as means ± standard deviations (SD) and absolute numbers. Statistical significance was assessed using Student’s *t* test for continuous variables and Pearson’s chi-squared test or Fisher exact test for categorical variables. Multiple logistic regression analysis was used to identify risk factors for immediate HS to cefaclor. *P* < 0.05 was considered statistically significant.

## Results

### Clinical characteristics of the study subjects

A total of 521 cefaclor ADRs were recorded during the study period. There were 19 and 502 cases of type A and B reactions, respectively, and there were no cases of severe cutaneous adverse reactions. Finally, 459 cases of cefaclor immediate HS were included in the present study to investigate the clinical characteristics of cefaclor-induced immediate HS. Of the 459 cefaclor immediate HS cases, 441 cases were prescribed cefaclor at other hospitals. The total number of exposed control cases in which no cefaclor ADR was detected by the pharmacovigilance centers, even though they had been prescribed cefaclor at the two study hospitals over the study period, was 20,183 (Fig. 1).

Table 2 depicts the demographic and clinical characteristics of the 459 immediate HSs to cefaclor and the 20,183 exposed controls. The proportion of females was significantly higher in the cefaclor immediate HS group than in the exposed control group (64.3% vs. 38.9%, *P* < 0.001), and cefaclor immediate HS cases were significantly younger than the exposed controls (46.8 ± 16.0 years vs. 50.4 ± 21.6 years, *P* < 0.001). The prevalences of hypertension (14.8% vs. 9.6%, *P* < 0.001), liver diseases (2.8% vs. 1.0%, *P* < 0.001), and allergic diseases, including asthma (6.8% vs. 0.7%, *P* < 0.001), allergic conjunctivitis (4.1% vs. 0.1%, *P* < 0.001), atopic dermatitis (2.0% vs. 1.1%, *P* = 0.063), urticaria (3.3% vs. 0.7%, *P* < 0.001), and food allergy (3.7% vs. 0.0%, *P* < 0.001), were greater in the cefaclor immediate HS group than in the exposed control group. Atopy was recorded in 40.4% of cases with cefaclor immediate HS. The number of concomitant medications was lower in the cefaclor immediate HS group than in the exposed control group (1.07 ± 1.1 vs. 1.84 ± 1.0, *P* < 0.001). Concomitant use of nonsteroidal anti-inflammatory drugs (NSAIDs) was significantly higher (42.5% vs. 28.7%, *P* < 0.001) in the cefaclor immediate HS group than in the exposed control group. However, the use of other concomitant medications, including other antibiotics (16.7% vs. 7.2%, *P* < 0.001), H2-blockers (32.6% vs. 10.2%, *P* < 0.001), gastrointestinal drugs (57.9% vs. 20.5%, *P* < 0.001), steroids (9.5% vs. 2.4%, *P* < 0.001), and antihistamines (20.5% vs. 9.4%, *P* < 0.001), was more frequently noted in the exposed control group than in the cefaclor immediate HS group. The most common indication for the use of cefaclor was respiratory system disease (KCD code: J00-J99) in both groups (Table 2).

**Table 2:** Clinical characteristics of cases in the cefaclor immediate HS and the exposed control groups

	Cefaclor immediate HS n = 459 (%)	Exposed control n = 20,183 (%)	*P*-value
Female	295 (64.3)	7848 (38.9)	< 0.001
Age (year)	46.8 ± 16.0	50.4 ± 21.6	< 0.001
≥ 65	59 (12.9)	5913 (29.3)	< 0.001
Underlying disease			
Diabetes mellitus	36 (7.8)	1713 (8.5)	0.623
Hypertension	68 (14.8)	1941 (9.6)	< 0.001
Liver diseases	13 (2.8)	208 (1.0)	< 0.001
Kidney diseases	13 (2.8)	679 (3.4)	0.531
Allergic disease			
Asthma	31 (6.8)	134 (0.7)	< 0.001
Allergic rhinitis and/or chronic sinusitis	96 (20.9)	4317 (20.9)	0.998
Allergic conjunctivitis	29 (4.1)	21 (0.1)	< 0.001
Atopic dermatitis	9 (2.0)	213 (1.1)	0.063
Urticaria	15 (3.3)	138 (0.7)	< 0.001
Food allergy	17 (3.7)	4 (0.0)	< 0.001
Atopy	80/198 (40.4)	N/A	
Number of concomitant medications	1.07 ± 1.1	1.84 ± 1.0	< 0.001
Concomitant drug use			
NSAIDs	295 (42.5)	5791 (28.7)	< 0.001
Other analgesics	54 (11.8)	2726 (13.5)	0.278
Other antibiotics	33 (7.2)	3362 (16.7)	< 0.001
Muscle relaxants	6 (1.3)	457 (2.3)	0.171
H2-blockers	47 (10.2)	6568 (32.6)	< 0.001
Gastrointestinal drugs	94 (20.5)	11,677 (57.9)	< 0.001
Steroids	11 (2.4)	1924 (9.5)	< 0.001
Antihistamines	43 (9.4)	4142 (20.5)	< 0.001
Most common indications for cefaclor prescription (KCD-code)			
1st	Respiratory system (J00-J99), 178 (38.8)	Respiratory system (J00-J99), 4270 (21.2)	
2nd	Digestive system (K00-J93), 65 (14.2)	Neoplasms (C00-D48), 3953 (19.6)	
3rd	Eye and adnexa (H00-H59), 34 (7.4)	Eye and adnexa (H00-H59), 2535 (12.6)	

*ADR* adverse drug reaction, *HS* hypersensitivity, *NSAID* nonsteroidal anti-inflammatory drug

Causality assessment of cefaclor immediate HS showed most of the cases to be of certain causality (42.5%). Cases were also evaluated based on serum specific IgE, skin tests, and/or oral provocation tests to cefaclor, as well as symptoms, signs, latency, drug history, and underlying conditions. Clinical manifestations of immediate HS included urticaria/skin rash (73.9%), angioedema (31.8%), dyspnea/hypoxemia (45.3%), hypotension (15.9%), altered mental status (6.3%), and anaphylaxis (61.2%). One patient died due to cardiac arrest as a result of anaphylaxis. Serum specific IgE to cefaclor was measured in 333 patients, and 181 (54.3%) of them had IgE levels of 0.35 kU/L or higher. A skin test to cefaclor was performed for a small sample of the 152 patients without specific IgE to cefaclor, and two were positive. An oral provocation test to cefaclor was performed for 22 patients, and all of them were positive (Table 3). In about half of the patients who underwent oral provocation test were accompanied by urticaria and angioedema in 10 patients. Hypoxemia occurred in one patient, and anaphylaxis in nine, but no patient died after oral provocation test.

**Table 3:** Clinical manifestations and immunologic evaluation of subjects with cefaclor immediate HS

	Cefaclor immediate HS n = 459 (%)
Causality	
Certain	195 (42.5)
Probable	111 (24.2)
Possible	153 (33.3)
Clinical manifestations	
Urticaria/skin rash	339 (73.9)
Angioedema	146 (31.8)
Dyspnea/hypoxemia	208 (45.3)
Hypotension	74 (15.9)
Altered mental status	29 (6.3)
Anaphylaxis	281 (61.2)
Serum specific IgE to cefaclor (+)	181/333 (54.3)
Skin test to cefaclor (+)	2/22 (9.1)
Provocation test to cefaclor (+)	22/22 (100)

### Risk factors for immediate hypersensitivity to cefaclor

Odds ratios (ORs) for cefaclor immediate HS relative to exposed controls were obtained by multiple logistic regression analyses (Table 4). Female sex (OR 2.917, 95% confidence interval [CI] 2.397–3.550, *P* < 0.001), age under 65 years (4.225, 3.017–5.916, *P* < 0.001), hypertension (2.520, 1.875–3.388, *P* < 0.001), liver diseases (2.189, 1.208–3.967, *P* = 0.010), asthma (8.075, 5.301–12.302, *P* < 0.001), and concomitant use of NSAIDs (1.888, 1.554–2.294, *P* < 0.001) were significantly associated with immediate HS to cefaclor (Table 4).

**Table 4:** Risk factors for immediate hypersensitivity to cefaclor by logistic regression analysis

	Univariate		Multivariate	
	Odds ratio (95% CI)	*P*-value	Odds ratio (95% CI)	*P*-value
Female	2.813 (2.319–3.412)	< 0.001	2.917 (2.397–3.550)	< 0.001
Age (year)	0.993 (0.988–0.997)	< 0.001	1.005 (0.999–1.011)	0.107
< 65	2.806 (2.132–3.695)	< 0.001	4.225 (3.017–5.916)	< 0.001
Underlying disease				
Hypertension	1.634 (1.257–2.122)	< 0.001	2.520 (1.875–3.388)	< 0.001
Liver diseases	2.754 (1.561–4.860)	< 0.001	2.189 (1.208–3.967)	0.010
Allergic disease				
Asthma	10.710 (7.167–16.003)	< 0.001	8.075 (5.301–12.302)	< 0.001
Concomitant drug				
NSAIDs	1.834 (1.521–2.213)	< 0.001	1.888 (1.554–2.294)	< 0.001

*CI* confidence interval, *NSAID* nonsteroidal anti-inflammatory drug

### Serologic biomarkers for immediate hypersensitivity and anaphylaxis to cefaclor

In the present study, we investigated several serologic indicators available as diagnostic markers for patients with cefaclor immediate HS. Serum levels of tryptase, total IgE, and specific IgEs to amoxicilloyl, ampicilloyl, penicilloyl G, and penicilloyl V did not significantly differ between patients with anaphylaxis and non-anaphylactic immediate HS to cefaclor. Serum levels of specific IgE to cefaclor were significantly higher in patients with anaphylaxis than in patients with non-anaphylactic immediate HS (6.45 ± 15.6 kU/L vs. 1.73 ± 4.7 kU/L, *P* = 0.004) (Table 5). The proportion of patients with high levels (> 0.35 kU/L) of specific IgE to cefaclor was also significantly higher in patients with anaphylaxis (153 in 236) than in patients with non-anaphylactic immediate HS (28 in 97) (64.8% vs. 28.9%, *P* < 0.001). In a prior study [5], we estimated the optimal cut-off values for specific IgE to cefaclor for anaphylaxis and immediate HS at 0.44 kU/L and 0.11 kU/L, respectively. Using the same cut-off values, the sensitivity and specificity for diagnosis of cefaclor anaphylaxis and immediate HS were, respectively, 64.0% and 76.0% for anaphylaxis and 65.6% and 65.6% for immediate HS.

**Table 5:** Serum levels of serological biomarkers in patients with anaphylaxis and non-anaphylactic immediate hypersensitivity to cefaclor

	Anaphylaxis (n = 281) Mean ± SD, (n)	Non-anaphylaxis (n = 178) Mean ± SD, (n)	*P*-value
Total IgE, kU/L	345 ± 509.88 (166)	331.28 ± 522.19 (64)	0.851
Tryptase, µg/L	16.36 ± 25.37 (62)	4.63 ± 2.75 (5)	0.309
Specific IgE to cefaclor, kU/L High cefaclor IgE (> 0.35 kU/L)	6.45 ± 15.62 (236) 153/236 (64.8%)	1.73 ± 4.69 (97) 28/97 (28.9%)	0.004 < 0.001
Specific IgE to amoxicilloyl, kU/L	0.12 ± 0.19 (131)	0.09 ± 0.09 (52)	0.302
Specific IgE to ampicilloyl, kU/L	0.22 ± 0.63 (127)	0.09 ± 0.10 (51)	0.155
Specific IgE to penicilloyl G, kU/L	0.17 ± 0.91 (126)	0.54 ± 3.18 (49)	0.237
Specific IgE to penicilloyl V, kU/L	0.38 ± 1.74 (122)	1.09 ± 6.86 (47)	0.285

*IgE* immunoglobulin E, *SD* standard deviation

## Discussion

Few studies have reported on the incidence rates of cefaclor hypersensitivity. In the study by Kammer et al., the incidence of cefaclor hypersensitivity was 1.1% among 3000 patients taking cefaclor [22]. Anaphylaxis to cefaclor, however, has been reported inconsistently for various populations [18, 23–25]. A retrospective study performed at a single-tertiary hospital in Korea showed that cefaclor was the most common culprit of drug-induced anaphylaxis [18]. A recent study using a database maintained by the German Federal Institute for Drugs and Medical Devices reported that cefaclor was the most common cause of antibiotics-induced anaphylaxis and the second most common cause of drug-induced anaphylaxis in children [25]. In the present study, we could not evaluate the incidence of cefaclor ADRs. However, cefaclor ADRs were mostly immediate HS reactions (88.1%), and more than half of them were anaphylaxis (53.9%). In Korea, antibiotics usage and prescription patterns from 2002 to 2013 were analyzed using the National Health Insurance Service-National Sample Cohort, and cefaclor was the most frequently prescribed cephalosporin, followed by cefuroxime and cephradine [26]. Cefaclor (9.0%) was the third most commonly prescribed antibiotic for upper respiratory infections from 2010 to 2012 in Beijing [23], but it was not listed as a commonly prescribed antibiotic from 2014 to 2017 in the United States [7–10]. This difference in the use and prescription of antibiotics among physicians and countries would affect the occurrence of cefaclor immediate HS and/or anaphylaxis.

In the present study, female sex, age under 65 years, hypertension, liver diseases, allergic diseases (e.g., asthma, allergic conjunctivitis, urticaria, and food allergy), and concomitant use of NSAIDs were significantly more common in patients with cefaclor immediate HS than in exposed controls. However, we identified underlying and allergic diseases using KCD codes. Thus, when interpreting our results, consideration should be given to the potential for mismatch between the actual disease and the given KCD code. Moreover, cases with cefaclor immediate HS were more likely to be evaluated for allergic disease in a more thorough manner than that in exposed controls. Nevertheless, the proportions of events accompanied by allergic rhinitis and/or chronic sinusitis did not differ between the two groups, because cefaclor was commonly prescribed for upper respiratory tract infections. Also, investigation of concomitant medications in cefaclor ADRs was somewhat incomplete, compared to that in exposed controls. This likely influenced the differences in concomitant drug use between them. However, concomitant use of NSAIDs, which would be associated with the occurrence of immediate HS, was significantly higher in cefaclor immediate HS cases.

Risk factors for drug hypersensitivity are largely divided into drug-related factors and host-related factors. Drug-related factors are primarily related with the chemical properties and molecular weight of a drug: other drug-specific risk factors include the dose, route of administration, duration of treatment, and frequency of exposure. Host-related factors include age, sex, atopy and atopic disease, genetic factors, and underlying diseases, such as infections or chronic diseases [3, 27, 28]. In the present study, risk factors were largely consistent with those described in previous studies investigating drug hypersensitivity, and the proportion of atopy was lower than that in other studies of non-steroidal anti-inflammatory drug intolerant patients [29, 30]. Moreover, we found that concomitant use of NSAIDs significantly increased the risk for cefaclor immediate HS, including anaphylaxis. NSAIDs have been suggested as a risk factor for anaphylaxis, particularly food-dependent exercise-induced anaphylaxis. This is presumed to be due to increased gastrointestinal tract permeability [31, 32]. However, there is no consensus on whether concomitant use of NSAIDs is a risk factor for drug-induced hypersensitivity or drug-induced anaphylaxis. In a study on 152 cases of in-hospital drug-induced anaphylaxis at a single-tertiary hospital in Korea, concomitant use of NSAIDs did not increase the likelihood of developing anaphylactic shock (OR 1.45, 95% CI 0.24–8.62, *P* = 0.686) [33].

Cephalosporin skin tests have not been standardized, and validated reagents are not present. Moreover, a proper cut-off value of serum specific IgE to cefaclor has not yet been validated. In the present study, 54.3% of the patients had serum specific IgE levels to cefaclor of 0.35 kU/L or higher, and only 9.1% exhibited positivity to cefaclor on a skin test in the cefaclor immediate HS group. However, all of the cases that underwent an oral provocation test to cefaclor in the cefaclor immediate HS group were positive, and thus, we think that the clinical diagnosis of cefaclor immediate HS in the present study was appropriate. There were only 22 patients who underwent both skin and oral provocation tests, and these tests were not possible to be conducted actively, due to problems with time, economic burden, risk of severe reactions, and patient refusal.

The present study had some limitations. First is the retrospective study design. Herein, we primarily analyzed spontaneously reported ADRs to cefaclor, and confirmation by immunologic evaluation was only performed in 42.5%. However, we investigated symptoms and signs related to ADRs and concomitant medications thoroughly, and included ADRs with possible causality or better. Second, there might be some cases in which ADRs related to cefaclor were not reported in the exposed control group. Third, this study was a multicenter study, and it is possible that the evaluation of the ADR cases at the two pharmacovigilance centers was not entirely congruent. However, expert allergists at both pharmacovigilance centers conducted a final review, and all reviewers discussed and made a final decision when any discrepancy was found in the assessment of ADRs. Fourth, we could not evaluate other risk factors, such as prior exposure history or exposed intensity of cefaclor immediate HS, because cefaclor is more commonly prescribed at primary care units than tertiary hospitals.

## Conclusions

We found that cefaclor elicited high proportions of immediate HS and anaphylaxis over the most recent 5 years. With the extensive use of cefaclor gradually increasing in Korea, it may be necessary to prepare a management plan for cefaclor immediate HS and anaphylaxis. Also, physicians ought to be cautious with prescribing cefaclor to females; individuals with hypertension, liver diseases, or asthma; and patients taking NSAIDs.

## Supplementary Information

**Additional file 1:** S1 description: A Standard Panel of 49 Allergen extracts for Skin Prick Tests

## Data Availability

The datasets used and/or analyzed during the current study are available from the corresponding author on reasonable request.

## Abbreviations

ADR: Adverse drug reaction
HS: Immediate hypersensitivity
KCD: Korean Standard Classification of Diseases
SD: Standard deviation
NSAID: Nonsteroidal anti-inflammatory drug
OR: Odds ratio
CI: Confidence interval
IgE: Immunoglobulin E
