# Supplementary material for: Clinical characteristics and risk factors for cefaclor-induced immediate hypersensitivity: a retrospective observation at two university hospitals in Korea
Source: Allergy Asthma Clin Immunol. 2021 Feb 15;17:20. doi: 10.1186/s13223-021-00523-8 (PMC7885353; doi:10.1186/s13223-021-00523-8)
Supplement: Supplementary file 1 — Additional file 1: S1 description: A Standard Panel of 49 Allergen extracts for Skin Prick Tests [file 13223_2021_523_MOESM1_ESM.rtf]

S1 description: A Standard Panel of 49 Allergen extracts for Skin Prick Tests

Skin prick tests (SPTs) (Allegropharma, Reinbek, Germany) with a standard panel of 49 allergen extracts were used in this study: Tree pollen mixture, Birch, Alder, Hazel, Beech, Ash, Elder, Oak, Poplar, Willow, Acasia, Elm, J. Cedar, Grass pollen mixture, Rye, Medow, Timothy, Orchard, Bermuda, Ragweed, Mugwort, Hop.J, Fat Hen, Nettle, Plantain, Dandelion, Alternaria, A. fumigatus, Cladosporium, Candida Albicans, Trychophyton, P. notatum, Mucor, Fusarium, A. niger, House dust mites (HDM) (Dermatophagoides farinae [Df], Dermatophagoides pteronyssinus [Dp], Tyrophagus putrescentiae [Tp]), Cat Fur, Dog Hair, Cockroach, Horse Hair, Cow Hair, Rabbit Hair, Pork Hair, Acarus siro, Rat epithelia, Hamster epithelia, Feathers, 10 mg/mL of histamine phosphate as a positive control, and 0.9% saline as a negative control. Most allergic disease clinics in Korea perform SPTs with 55 allergens, and most of the allergens are similar to those listed above.[1]

Reference
1.	Lee JE, Ahn JC, Han DH, Kim DY, Kim JW, Cho SH, et al. Variability of offending allergens of allergic rhinitis according to age: optimization of skin prick test allergens. Allergy Asthma Immunol Res. 2014;6(1):47-54.
